# Supplementary material for: Stability, Morphology, and Effects of In Vitro Digestion on the Antioxidant Properties of Polyphenol Inclusion Complexes with β-Cyclodextrin
Source: Molecules. 2022 Jun 14;27(12):3808. doi: 10.3390/molecules27123808 (PMC9228204; doi:10.3390/molecules27123808)
Supplement: Supplementary file 1 [file molecules-27-03808-s001.zip › molecules-1747396-supplementary.pdf]

Supplementary material

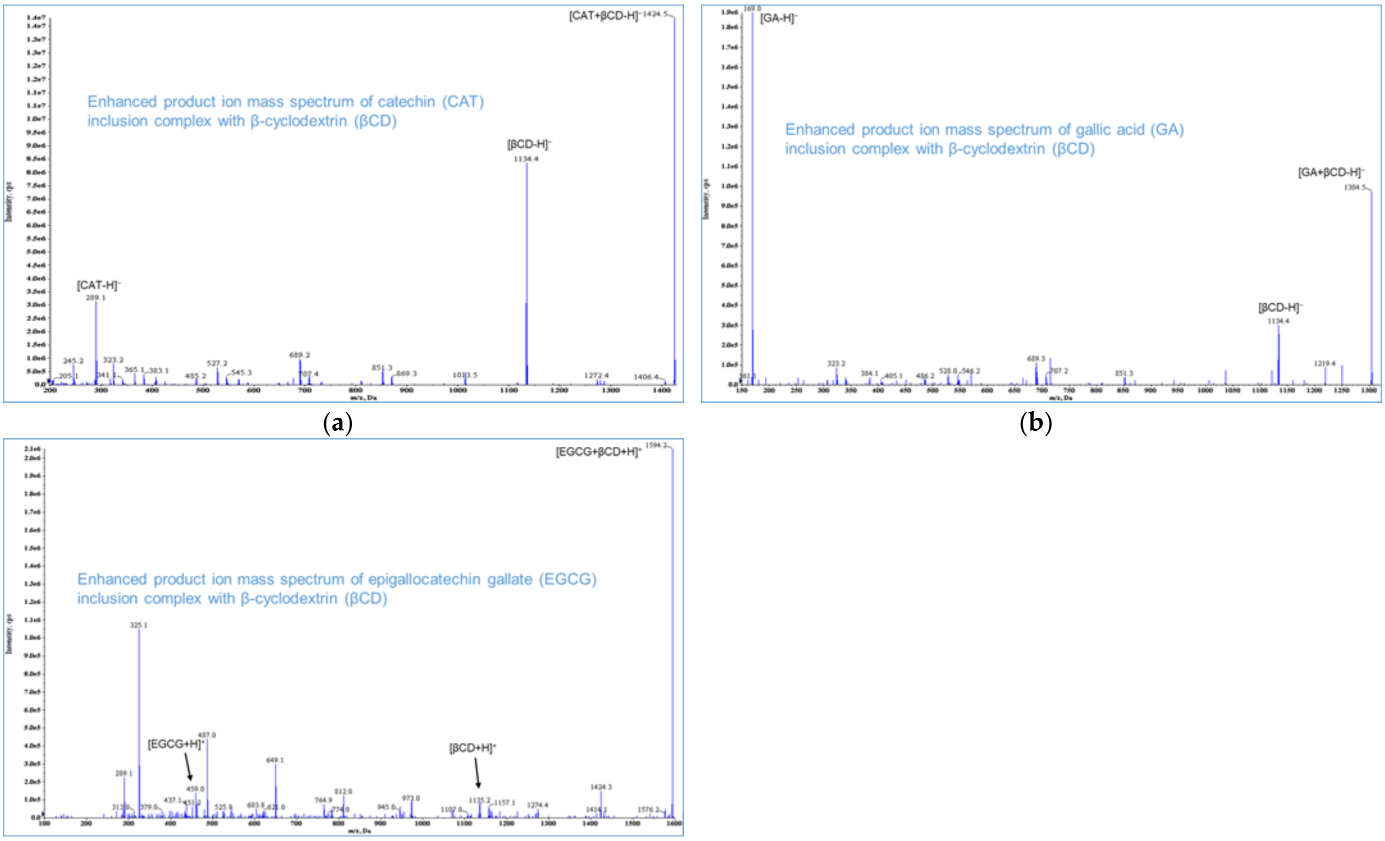

(**a**) (**b**) (**c**)

Figure S1. Enhanced product ions scan mass spectrum of βCD inclusion complexes with (**a**) CAT, (**b)** GA and (**c**) EGCG. βCD – beta cyclodextrin, CAT – catechin, GA – gallic acid, EGCG – epigallocatechin gallate.

Table S1. Polyphenol-polyphenol interactions on antioxidant activity (ORAC assay (μM TE)).

	**CAT/GA**	**CAT/EGCG**	**GA/EGCG**	**CAT/GA/EGCG**
		**Non-encapsulated**		
**Expected**	23.53 ± 1.234	31.97 ± 2.06	29.41 ± 1.56	42.46 ± 2.36
**Observed**	22.25 ± 0.36	20.44 ± 1.03*	23.14 ± 0.30*	23.35 ± 1.14*
**p value**	0.14	< 0.001	< 0.001	< 0.001
**Interaction**	Additive	Antagonistic	Antagonistic	Antagonistic
		**Encapsulated**		
**Expected**	24.11 ± 1.11	33.52 ± 1.38	30.58 ± 1.56	30.58 ± 1.56
**Observed**	22.56 ± 0.40	21.87 ± 0.99*	22.54 ± 0.39*	24.03 ± 1.01*
**p value**	0.07	< 0.001	< 0.001	< 0.001
**Interaction**	Additive	Antagonistic	Antagonistic	Antagonistic

CAT – catechin, GA – gallic acid, EGCG – epigallocatechin gallate, μM TE – micromolar Trolox equivalents. The * denotes significant ($p < 0.05$) difference between the expected and observed values in the same column for non-encapsulated and encapsulated samples. The data is represented as mean ± SEM of at least five experiments done in duplicates.

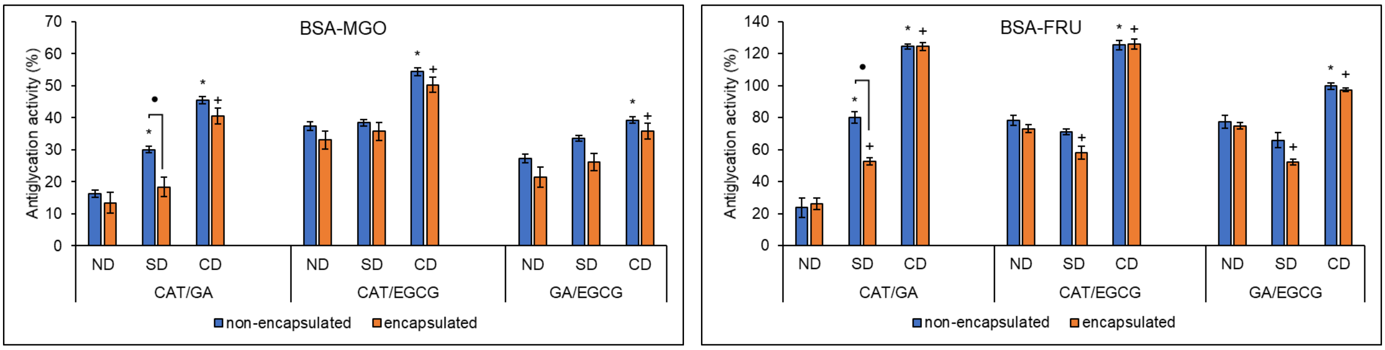

(**a**) (**b**)

Figure S2. Inhibition of AGEs formation by non-digested (ND), and following simple (SD) and com-plex (CD) digestion of non- and encapsulated CAT/GA, CAT/EGCG, and GA/EGCG combinations evaluated with the (**a**) BSA-MGO and (**b**) BSA-FRU models at 100 μM for each polyphenol in each sample. The data is represented as mean ± SEM of at least five experiments done in duplicates. ND – non-digested, SD – simple digestion, CD – complex digestion, AGEs – advanced glycation end-products, BSA – bovine serum albumin, MGO – methylglyoxal, FRU – fructose, CAT – catechin, GA – gallic acid, EGCG – epigallocatechin gallate. The * and + represent significant ($p < 0.05$) differences between non- and encapsulated ND compared with respective SD or CD samples. The ● denotes significant ($p < 0.05$) difference between the non- and encapsulated for each treatment.

Table S2. Polyphenol-polyphenol interactions on antiglycation activity (AGEs assay (%)).

**BSA-MGO**	**CAT/GA**	**CAT/EGCG**	**GA/EGCG**	**CAT/GA/EGCG**
		**Non-encapsulated**		
**Expected**	16.64 ± 2.55	44.85 ± 2.65	23.59 ± 2.42	42.54 ± 3.75
**Observed**	16.26 ± 1.21	37.36 ± 1.28*	27.31 ± 1.29*	38.02 ± 1.30*
**p value**	0.71	< 0.001	0.01	0.04
**Interaction**	Additive	Antagonistic	Synergistic	Antagonistic
		**Encapsulated**		
**Expected**	8.28 ± 7.27	34.20 ± 7.11	17.47 ± 7.22	29.98 ± 10.78
**Observed**	13.42 ± 3.34	33.06 ± 2.85	21.39 ± 3.18	33.63 ± 2.75
**p value**	0.10	0.72	0.21	0.54
**Interaction**	Additive	Additive	Additive	Additive

**BSA-FRU**	**CAT/GA**	**CAT/EGCG**	**GA/EGCG**	**CAT/GA/EGCG**
		**Non-encapsulated**		
**Expected**	41.64 ± 11.12	≥ 100.00	88.17 ± 9.65	≥ 100.00
**Observed**	23.77 ± 6.15*	78.15 ± 3.09*	77.39 ± 3.96*	80.84 ± 4.73*
**p value**	0.001	< 0.001	0.03	< 0.001
**Interaction**	Antagonistic	Antagonistic	Antagonistic	Antagonistic
		**Encapsulated**		
**Expected**	48.34 ± 5.64	≥ 100.00	89.78 ± 4.11	≥ 100.00
**Observed**	26.30 ± 3.63*	73.05 ± 2.49*	74.88 ± 2.16*	75.42 ± 1.57*
**p value**	< 0.001	< 0.001	< 0.001	< 0.001
**Interaction**	Antagonistic	Antagonistic	Antagonistic	Antagonistic

CAT – catechin, GA – gallic acid, EGCG – epigallocatechin gallate, AGEs -advanced glycation end-products, BSA – bovine serum albumin, MGO – methylglyoxal, FRU – fructose. The * denotes significant ($p < 0.05$) difference between the expected and observed values in the same column for non-encapsulated and encapsulated samples. The data is represented as mean ± SEM of at least five experiments done in duplicates.

Table S3. Polyphenol-polyphenol interactions on cellular antioxidant activity (DCFH-DA assay (%)).

[10 µM]	CAT/GA[+]	CAT/EGCG	GA[+]/EGCG	CAT/GA[+]/EGCG
	**Non-encapsulated**			
**Expected**	30.68 ± 11.13	58.63 ± 16.53	27.94 ± 8.25	58.63 ± 16.53
**Observed**	36.73 ± 8.22	69.51 ± 4.44	73.03 ± 2.81*	75.60 ± 3.69
**p value**	0.39	0.26	< 0.001	0.09
**Interaction**	Additive	Additive	Synergistic	Additive
	**Encapsulated**			
**Expected**	21.15 ± 11.78	44.46 ± 21.31	23.30 ± 12.13	44.46 ± 21.31
**Observed**	43.15 ± 7.45*	68.73 ± 5.45	67.97 ± 6.22*	80.20 ± 3.30*
**p value**	0.001	0.07	< 0.001	0.02
**Interaction**	Synergistic	Additive	Synergistic	Synergistic

[100 µM]	CAT/GA	CAT/EGCG	GA/EGCG	CAT/GA/EGCG
	**Non-encapsulated**			
**Expected**	≥ 100.00	≥ 100.00	≥ 100.00	≥ 100.00
**Observed**	93.34 ± 0.78	98.46 ± 0.62	101.62 ± 0.88	99.10 ± 0.49
**Interaction**	Additive	Additive	Additive	Additive
	**Encapsulated**			
**Expected**	≥ 100.00	≥ 100.00	≥ 100.00	≥ 100.00
**Observed**	95.75 ± 1.39	98.91 ± 0.71	102.14 ± 0.68	100.58 ± 0.57
**Interaction**	Additive	Additive	Additive	Additive

CAT – catechin, GA – gallic acid, EGCG – epigallocatechin gallate. The * denotes significant ($p < 0.05$) difference between the expected and observed values in the same column for non-encapsulated and encapsulated samples. +Gallic acid had no quantifiable activity.
